# Tripartite signalling by NMDA receptors

**DOI:** 10.1186/s13041-020-0563-z

**Published:** 2020-02-18

**Authors:** Vishaal Rajani, Ameet S. Sengar, Michael W. Salter

**Affiliations:** 1 Program in Neurosciences & Mental Health, Hospital for Sick Children, Toronto, Ontario M5G 1X8 Canada; 2 Department of Physiology, University of Toronto, Toronto, Ontario M5S 1A8 Canada

**Keywords:** NMDA receptors, Synaptic plasticity, Ionotropic, Metabotropic, Signalling cascades, Protein kinases

## Abstract

*N*-methyl-d-aspartate receptors (NMDARs) are excitatory glutamatergic receptors that are fundamental for many neuronal processes, including synaptic plasticity. NMDARs are comprised of four subunits derived from heterogeneous subunit families, yielding a complex diversity in NMDAR form and function. The quadruply-liganded state of binding of two glutamate and two glycine molecules to the receptor drives channel gating, allowing for monovalent cation flux, Ca^2+^ entry and the initiation of Ca^2+^-dependent signalling. In addition to this ionotropic function, non-ionotropic signalling can be initiated through the exclusive binding of glycine or of glutamate to the NMDAR. This binding may trigger a transmembrane conformational change of the receptor, inducing intracellular protein-protein signalling between the cytoplasmic domain and secondary messengers. In this review, we outline signalling cascades that can be activated by NMDARs and propose that the receptor transduces signalling through three parallel streams: (i) signalling via both glycine and glutamate binding, (ii) signalling via glycine binding, and (iii) signalling via glutamate binding. This variety in signal transduction mechanisms and downstream signalling cascades complements the widespread prevalence and rich diversity of NMDAR activity throughout the central nervous system and in disease pathology.

## Introduction

NMDA receptors (NMDARs) are excitatory glutamatergic receptors that are found throughout the mammalian central nervous system (CNS) and are fundamental to many neuronal processes. Native NMDARs are tetrameric assemblies, typically made up of two GluN1 subunits, and two GluN2 subunits. GluN3 subunits, which can form excitatory glycine channels with GluN1 [1], are not the focus of this review. GluN2 subunits are encoded by four genes, *Grin2A-D,* whereas GluN1 is encoded by a single gene, *Grin1*, that can undergo alternative splicing to form eight variants [2]. The arrangement of these subunits to form a tetramer is critical to ion channel function and imparts NMDARs with a rich diversity in ionotropic modulation, channel kinetics, mobility, and signal transduction [3].

Cryo-electron microscopy and X-ray crystallography have revealed the tetrameric assembly of GluN1 and GluN2 subunits as a dimer of dimers, with alternating subunits around the ion pore [4–8]. The receptor assembly is comprised of four structural layers: the amino-terminal domain (NTD), the agonist-binding domain (ABD), the transmembrane domain (TMD) and the intracellular C-terminal domain (CTD). The NTD has a clamshell-like structure and is involved in allosteric regulation. The agonist binding domain binds glycine and d-serine (GluN1) and glutamate (GluN2) to drive opening of the ion pore which is formed by the TMDs. The CTD is important for stabilization via binding to scaffolding proteins, trafficking via lateral diffusion or endocytosis, and signalling through phosphorylation by a number of second messengers. Thus, each domain allows for the physiological function of the NMDAR and for ionotropic activity to be modulated in several ways.

**Fig. 1:**
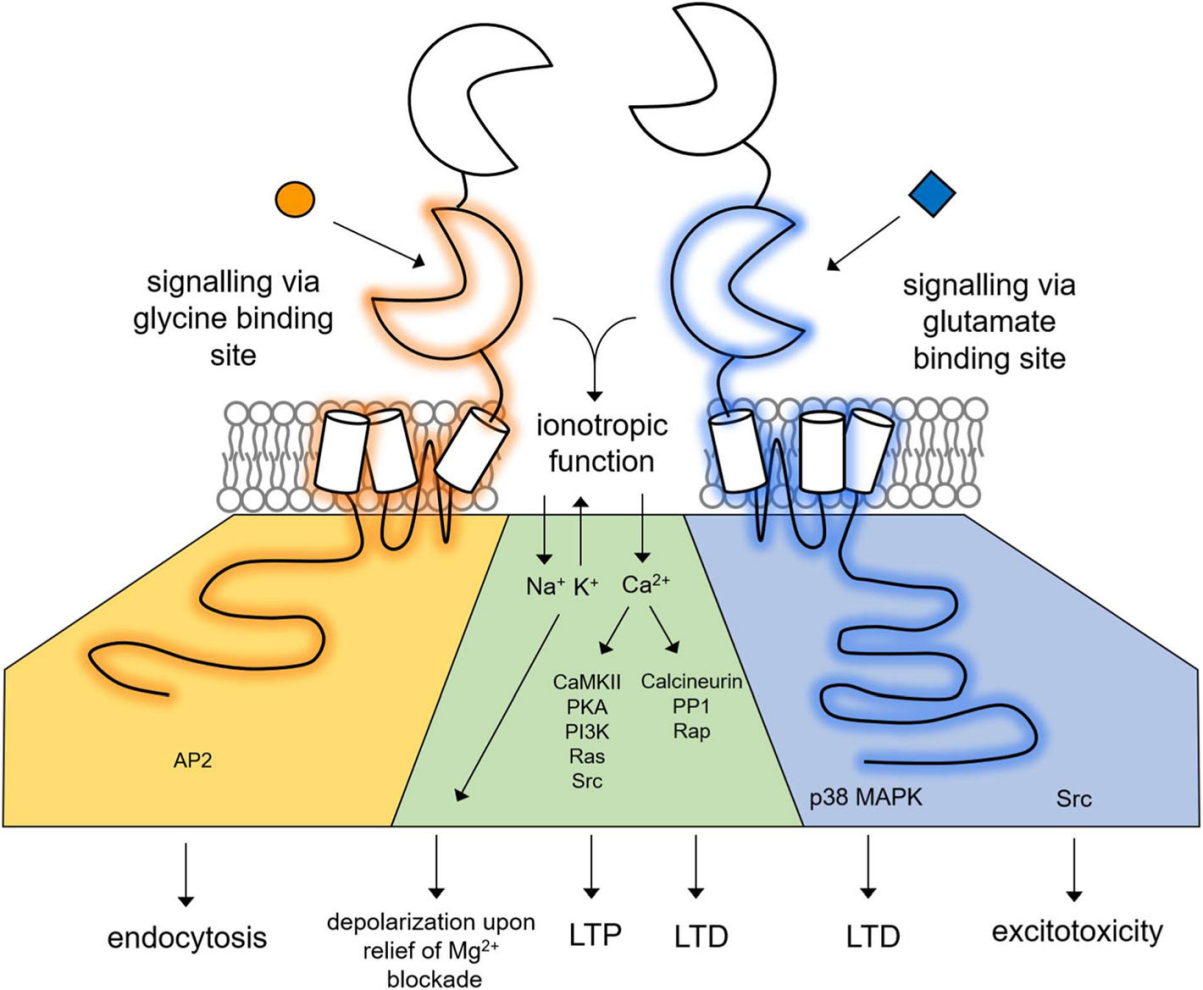
Tripartite signalling of the NMDAR. A hypothesized model by which the NMDAR transduces signals in three parallel streams. The binding of glycine and glutamate to the ABD mediate channel gating and ionotropic function causing depolarization through monovalent cation flux and through calcium influx to downstream calcium-dependent pathways. The NMDAR can also signal non-ionotropically, through either glycine or glutamate binding independent of binding of the other co-agonist, initiating conformational changes propagated across the plasma membrane, and downstream protein-protein interactions

However, accumulating evidence of non-ionotropic functions of NMDARs is shifting the current paradigm of the receptor solely as a ligand-gated ion channel to that of a dynamic signalling macromolecule capable of not only ionotropic but also non-ionotropic function. The non-ionotropic functions of NMDARs are mediated through ligand binding to the extracellular ABD which is hypothesized to induce conformational changes that are transduced across the cell membrane to effect changes in the conformation of the intracellular CTD. These changes initiate downstream signalling cascades via protein-protein interactions with some of the many intracellular mediators associated with the NMDAR macromolecule. Here, we propose a framework of the NMDAR as a tripartite signalling receptor complex, that can transduce, compute and transmit information through three parallel streams (i) signalling via the binding of both co-agonists glutamate and glycine to the receptor, (ii) signalling via exclusive glycine binding, and (iii) signalling via exclusive glutamate binding (Fig. 1). This framework outlines the distinctive signalling roles of NMDARs in the context of normal synaptic transmission, cognitive processes, and targetable mechanisms underlying disease. Compounded by the diversity in subunits, this previously unanticipated richness in signalling matches the prevalence of the receptor in a multitude of neurological functions and disorders.

### NMDAR signalling via binding glutamate and glycine

Canonical NMDAR signalling is mediated through its ionotropic function initiated by binding of two molecules of each of the co-agonists glycine (or d-serine) and glutamate. Binding of these co-agonists produces conformational changes in the extracellular domains of the NMDAR which are transduced to opening of the ion channel conductance pathway (i.e. the ‘pore’), allowing selective permeability to cations, including Na^+^, K^+^ and Ca^2+^. The permeability of the NMDAR pore to the predominant intracellular and extracellular monovalent cations – K^+^ and Na^+^, respectively – results in depolarization from the normal resting membrane potential of CNS neurons. Under basal physiological conditions this NMDAR-induced depolarization is minimized because of strong inhibition, often erroneously called ‘block’, of current flow through the pore by magnesium. Magnesium permeates, but sticks within, the pore and transitions much more slowly than Na^+^ or K^+^. The inhibition of current flow by magnesium produces a region of ‘negative slope conductance’ in the current-voltage relationship [9] which allows small, repeated depolarizations of the membrane potential caused by NMDARs to feed-forward producing phenomena such as ‘windup’ of neuronal firing [10]. NMDAR-mediated depolarizations are also increased by relief of magnesium inhibition when the membrane potential is otherwise depolarized by excitatory synaptic inputs and firing activity [11] or by suppression of resting K^+^ conductances by G-protein-coupled receptors [12].

In contrast to the fast basal excitatory signalling of AMPA receptors, NMDARs are susceptible to magnesium inhibition at negative potentials, and are equipped with a high calcium permeability, placing them in a unique position as molecular coincidence detectors to initiate calcium-dependent signalling cascades. Indeed, NMDARs can be a significant source of cytosolic free calcium, which is critical to synaptic long-term potentiation (LTP). In the hippocampus, a high frequency stimulation of Schaffer collateral input to CA1 neurons causes a large influx of calcium through NMDARs, leading to the activation of a number of kinases and the downstream insertion of AMPA receptors into the synapse [13]. Most notable among these kinases is calcium/calmodulin kinase II (CaMKII), which upon activation, translocates to the post-synaptic density (PSD) to form a CaMKII/NMDAR complex [14]. NMDAR dependent LTP is susceptible to pharmacological block of CaMKII [15], and is deficient in CaMKII genetic knock out mice [16], suggesting that the activation of this kinase is necessary for LTP induction. Moreover, inhibition of the CaMKII/NMDAR complex can reduce the long term potentiation effect, suggesting that formation of the complex acts like a molecular ‘switch’ to maintain synaptic strength [14].

NMDAR-dependent long-term depression (LTD) of hippocampal CA1 synapses, on the other hand, can be induced by a low frequency stimulation of Schaffer collaterals, and requires a more gradual increase in intracellular calcium through activation of NMDARs. This synaptic depression involves the activation of a number of phosphatases through NMDAR calcium entry, including the calcium/calmodulin dependent phosphatase, calcineurin. Associated with the endocytic machinery dynamin/amphiphysin, calcineurin acts as a calcium sensor to initiate endocytosis of AMPA receptors [17]. Indeed, NMDAR mediated AMPA receptor internalization is susceptible to intracellular calcium chelators, as well as calcineurin inhibitors [18], suggesting a significant role for calcineurin in NMDAR-dependent LTD. Calcineurin has also been suggested to activate downstream protein phosphatase 1 (PP1), which has mixed effects on AMPA receptor trafficking [18, 19].

In addition to calcium entry, NMDAR mediated sodium influx may also initiate downstream signalling cascades. High frequency stimulation can induce sodium concentrations of up to 100 mM in active spines, which can be inhibited by NMDAR blockade [20]. Intracellular sodium increases can cause amplification of NMDAR currents and single channel activity in cultured spinal and hippocampal neurons, suggesting a role for sodium in controlling the gain of excitatory synaptic transmission [21]. This sensitivity to sodium is suggested to be set by Src tyrosine kinase and can overcome calcium-dependent inactivation of NMDARs [22–24].

Glycine and glutamate co-agonist binding to the ABD of the NMDAR can initiate a variety of signalling cascades through ionotropic function, mediated by depolarization, and by Ca^2+^ and Na^+^ entry. These signalling pathways can produce opposing physiological outcomes, and is complicated by the dynamic changes in subunit expression, which change during development and in a number of cognitive disorders [3].

### NMDAR signalling via glycine binding only

In addition to signalling initiated by simultaneous occupancy of the glutamate and the glycine binding sites, there is evidence that NMDARs are also capable of signalling by binding to the glycine or the glutamate binding site independent of the other site. The earliest evidence of this signalling was demonstrated via glycine stimulation of the receptor independent of the glutamate site [25], priming the receptors for a use-dependent receptor internalization. In HEK293 cells, it was found that NMDA and glycine stimulation may cause a decline in peak current of GluN1/GluN2A recombinant NMDARs [26] but whether this signalling requires the binding of one or both ligands to the receptor was not examined. In isolated hippocampal neurons, a similar use-dependent decline in NMDAR-mediated currents was observed when NMDA-glycine stimulation of the receptors, to evoke currents, were preceded by a glycine conditioning stimulus [25]. This decline in current was reduced by inhibitors of dynamin-dependent endocytosis, suggesting that glycine stimulation primes receptors for endocytosis, resulting in a decrease in cell surface receptors. Co-immunoprecipitation following glycine stimulation showed increased association of the NMDAR with a principal component of the intracellular endocytic adaptor protein, AP2, identifying the activation of a downstream endocytic pathway. This association persisted when glycine stimulation was applied in the presence of a glutamate binding site antagonist d-APV, but was blocked by the glycine-site competitive inhibitors indicating that glycine site stimulation alone is sufficient to prime the endocytic process [25, 27]. The receptors are subsequently internalized by glycine and glutamate stimulation but this also appears to be independent of ion flux as endocytosis is resistant to blocking the pore with MK-801 (unpublished observations). Moreover, the internalization of functional channels shown by cell ELISA (enzyme linked immunosorbent assay) is markedly reduced in extracellular hypertonic sucrose solution, restricting clathrin-dependent endocytosis [25].

The priming of NMDARs for endocytosis by glycine suggests that a higher concentration of glycine, beyond the normal saturating concentration required for ionotropic function, can activate an alternative signalling pathway, independent of ion flux. The capacity of the NMDAR to signal in two different ways via glycine binding could be explained by the existence of two affinities at the glycine binding site: a high affinity for receptor gating, and a low affinity for receptor priming. Indeed, a second lower affinity at the glycine site has been previously reported [28–30]. The alternative possibility is that glycine binding yields divergent effects based on different coupling gains [31]. In this scenario, glycine binding could give rise to two different concentration-response relationships; a “high gain” concentration-response relationship that controls gating, and a “low gain” relationship at higher glycine concentrations which primes receptors for internalization. These responses are mediated by the same ligand acting on the same binding site of the receptor, but the high-gain effect is nearly saturated even at concentrations which just begin to elicit the lower gain effect. Further characterization of these mechanisms is ongoing.

Basal extracellular glycine and d-serine levels are typically in the range of 5–10 μM, but vary based on brain region. Areas such as the cerebellum and prefrontal cortex have higher (> 20 μM) basal levels of extracellular glycine, while others, such as the striatum, have lower levels of glycine, but higher d-serine levels [32]. Based on these observations, basal glycine and d-serine levels normally sit just below the ‘set point’ of glycine priming. As a result, an increase in extracellular glycine or d-serine levels could signal the initiation of receptor internalization, and this may be an important factor in determining the basal stability of cell surface NMDARs. Glycine priming may also be significant for controlling synaptic signalling in the presence of allosteric modulators that change the potency of glycine/d-serine binding to GluN1 [32]. In addition, glycine priming may be functionally important for changes to glycine and d-serine signalling, which may mediate the migration of receptors between synaptic and extrasynaptic compartments [33], or during developmental changes in subunit composition [34]. Glycine levels also increase in many different pathological conditions such as brain trauma, ischemia, or epilepsy [32], where glycine priming could act as a homeostatic mechanism to remove functional NMDARs and prevent excitotoxic or neurotoxic signalling cascades. Following internalization, NMDA receptors may be targeted for degradation, recycled and reinserted at the cell surface, or may in fact initiate a downstream signalling cascade to activate protein kinase D1 (PKD1) to modify the signalling of non-internalized receptors [35, 36].

Glycine-primed internalization was the first observation to suggest the possibility of a transmembrane signalling process by which NMDAR agonist binding could produce intracellular conformational changes to initiate biochemical signalling, independent of ion flux. This response to glycine signalling sets the precedence for other types of non-ionotropic signalling mediated by ligand binding to the receptor. Further understanding of the physiological context of this type of signalling will depend on factors such as NMDAR subunit composition, cell type, and receptor localization.

### NMDAR signalling via glutamate binding only

In addition to the non-ionotropic signaling by the glycine site there is evidence that agonist binding to the glutamate binding site can initiate metabotropic signalling. Specifically, activating GluN2 has been shown to initiate non-ionotropic signalling resulting in a form of LTD [37]. Low-frequency stimulation induced LTD which was blocked by d-APV, was produced in the presence of the NMDAR ion-channel blocker MK-801 and the glycine site antagonist, 7-CK, indicating that ligand binding to the glutamate binding site on GluN2 is sufficient to produce LTD in hippocampal slice preparations [37]. Moreover, low-frequency glutamate stimulation when the glycine site or pore are blocked also induces structural plasticity of dendritic spines, causing spine shrinkage in the absence of a strong calcium influx [38]. The underlying mechanism of this synaptic weakening involves the downstream activation of p38 MAPK, which is implicated in AMPAR trafficking [39] and in the cofilin-mediated cytoskeletal changes necessary for structural dendritic changes [40]. These observations contrast with the common view that low levels of calcium entry are necessary to induce LTD [41].

The significance of this form of NMDAR signalling also extends to disease pathology. Parallel findings suggest that amyloid beta induced synaptic depression in hippocampal slice cultures is not dependent on NMDAR ion flux [42], but rather through a d-APV sensitive, and GluN2B selective process, effecting a GluN2B to GluN2A subunit switch [43], and p38 MAPK-mediated synaptic loss [44]. Excitotoxic amounts of NMDA have been reported to cause an initial current through the receptor and a secondary current through pannexin-1, mediated through the NMDAR activation of Src kinase [45]. Additional findings suggest that while the initial excitotoxic NMDA induced current can be blocked by MK-801, the secondary current persists, resulting in dendritic ‘blebbing’, calcium dysregulation, mitochondrial dysfunction and cell death [46]. In this case, while high NMDA concentration seems to be the main mediator of Src activation, both the pannexin-1 mediated current and dendritic blebbing were blocked by antagonists of either glutamate (d-APV) or glycine (CGP-78608) binding sites suggesting that both are required to carry out this pathway [46]. These observations argue a role for non-ionotropic signalling of NMDARs in the pathophysiology of Alzheimer’s disease and ischemic injury and may present alternate strategies for treatment of neurodegenerative diseases or cognitive impairment, in targeting signalling pathways without affecting normal ionotropic function.

### Outstanding questions

A major unresolved question is how ligand binding to either the glycine or the glutamate site alone is transduced within the extracellular domains of NMDAR complex. This question has been addressed in part for the priming of the receptor complex by glycine. Investigation of the molecular determinants of glycine-primed internalization has revealed that recombinant NMDARs containing GluN2A or GluN2B equally respond to glycine priming, measured via decline in whole cell currents, increased association with AP2, and fluorescent imaging of internalized NMDARs [47]. A point mutation A714L on GluN1, when expressed together with either GluN2A or GluN2B, has been found to abolish glycine priming in recombinant HEK cells, without affecting ion pore opening [47]. Moreover, NMDARs with GluN1 splice variants lacking the N1 cassette in the ATD, are primed by glycine whereas receptors containing the N1 cassette are not [48]. Both N1-containing and N1-lacking NMDARs, however, gate normally upon co-agonist stimulation [49]. Together these findings indicate that the molecular requirements within the extracellular region of GluN1 for glycine-induced priming differ from those for co-agonist gating. Determining whether there are differing molecular constraints within the extracellular domains of GluN2 subunits, or elsewhere in the extracellular parts of the NMDAR complex, that are necessary for non-ionotropic versus ionotropic signalling resulting from glutamate binding, remains to be determined. Recent findings suggest that ligand binding to the glycine site of GluN1 may initiate non-ionotropic signalling in a GluN2A-specific manner [50]. However, the signalling initiated by glycine that primes NMDARs for internalization is not GluN2-subunit specific [47], suggesting that particular NMDAR tetrameric configurations may allow for GluN1-GluN2 subunit interactions to initiate certain types of downstream signalling but not others. Glycine may, in addition to binding to GluN1, bind to GluN3, forming excitatory glycine receptors [1, 51]. Whether GluN1/GluN3 receptors can signal non-ionotropically has not yet been explored.

Another unresolved question is whether there are structural changes within the intracellular domains of the NMDARs that are initiated by single-ligand binding. That ligand binding may initiate transmembrane signalling from the ABD to the CTD, triggering changes in intracellular protein-protein interactions is supported by the use of fluorescence lifetime imaging and fluorescence resonance energy transfer which have detected the movement of GluN1 cytoplasmic domains in response to extracellular GluN2 binding, in the presence of MK-801 and 7-CK [52]. The details of the intracellular conformational change will require further structural modelling to determine how the movement of the cytoplasmic domain rests in the current paradigm of the allosteric ‘rolling’ interactions between the NTD and the ABD within the receptor [53].

In this review, the non-ionotropic signalling upon which we focused is that mediated through binding of either glutamate or glycine to the NMDAR. Although it has yet to be observed, we cannot exclude the possibility that there are alternate non-ionotropic signalling pathways that require the binding of both ligands for initiation. For example, alternate NMDAR signalling pathways have been suggested to induce intracellular calcium increases in cultured astrocytes, although it is not clear whether both binding sites is required for this metabotropic function [54, 55].

Implicit within the above explanations for non-ionotropic NMDAR signalling is that the signalling is via heterometric receptor protein complexes. However, as NMDARs are dimers of heterodimers it is conceivable that monomers or heterodimers might exist on the cell surface. Such heterodimers would not be capable of forming pores, which requires tetramers, and would be electrically ‘silent’, but would still have GluN1 and GluN2 subunits capable of binding glycine and glutamate, respectively, and thus could signal non-ionotropically. We wonder whether it is such GluN1/GluN2 heterodimers, or even GluN1 monomers themselves (see [56]) that are responsible for the non-ionotropic signalling described above. This explanation may appear fanciful but recent data suggest that AMPARs are in fact ‘metastable’ within the plasma membrane and can quickly transition to monomers and dimers, only to readily form tetramers again [57]. The exclusion of NMDAR ionotropic function removes the requirement for a tetrameric structure, so it is not unreasonable to consider that NMDAR subunits, existing as monomers or heterodimers on the cell surface could signal via the non-ionotropic transmembrane conformational change as one would conceive for a heterotetrameric NMDAR.

## Summary

The findings presented in this review suggest that the NMDAR is capable of sensing and distinguishing between a variety of extracellular and intracellular conditions to produce, via tripartite signalling, often opposite, physiological outcomes. These outcomes likely depend on membrane depolarization to remove endogenous Mg^2+^ block, the availability of intracellular signalling partners, synaptic and extrasynaptic cellular localization, and the brimming diversity of subunits which make up the tetramer. As with current models of NMDAR function, based on crystallography, experimental, and in silico advances, investigation of this complex problem will require a macromolecular approach, involving not only the interaction between subunits, but also the interacting domains of the receptor. Understanding the dominance, interaction and control of these signalling streams is key to understanding disease pathology in NMDAR-centric disorders, and the strategic development of therapeutics to target specific pathways without affecting normal function.

## Data Availability

Not applicable.

## Abbreviations

7-CK: 7-chlorokynurenate, glycine-site antagonist
ABD: Agonist binding domain
AMP: Adenosine monophosphate
AMPAR: ɑ-amino-3-hydroxy-5-methyl-4-isoxazolepropionic acid receptor
AP2: Endocytic adaptor protein 2
BAPTA: (1,2-bis(o-aminophenoxy)ethane-N,N,N′,N′-tetraacetic acid
Ca^2+^: Calcium
CAMKII: Calcium/calmodulin kinase II
CGP-78608: Glycine-binding site antagonist
CTD: C-terminal domain
d-APV: Glutamate-binding site antagonist
FLIM: Fluorescence lifetime imaging
FRET: Fluorescence resonance energy transfer
GluN1: Glycine-binding NMDA receptor subunit 1
GluN2: Glutamate-binding NMDA receptor subunit 2
GluN3: Glycine-binding NMDA receptor subunit 3
GTP: Guanosine triphosphate
K^+^: Potassium
LTD: Long-term depression
LTP: Long-term potentiation
MAPK: Mitogen-activated protein kinase
MK-801: Ion pore blocker
Na^+^: Sodium
NMDAR: N-methyl-D-aspartate receptor
NTD: Amino-terminal domain
PI3K: Phosphatidylinositol 3 kinase
PKA: Protein kinase A
PSD: Post-synaptic density
SFK: Src family kinase
TMD: Transmembrane domain

## References

[1] Grand T, Abi Gerges S, David M, Diana MA, Paoletti P (2018). Unmasking GluN1/GluN3A excitatory glycine NMDA receptors. Nat Commun.

[2] Hollmann M, Boulter J, Maron C, Beasley L, Sullivan J, Pecht G (1993). Zinc potentiates agonist-induced currents at certain splice variants of the NMDA receptor. Neuron..

[3] Paoletti P, Bellone C, Zhou Q (2013). NMDA receptor subunit diversity: impact on receptor properties, synaptic plasticity and disease. Nat Rev Neurosci.

[4] Furukawa H, Gouaux E (2003). Mechanisms of activation, inhibition and specificity: crystal structures of the NMDA receptor NR1 ligand-binding core. EMBO J.

[5] Lee CH, Gouaux E (2011). Amino terminal domains of the NMDA receptor are organized as local heterodimers. PLoS One.

[6] Lee CH, Lu W, Michel JC, Goehring A, Du J, Song X (2014). NMDA receptor structures reveal subunit arrangement and pore architecture. Nature..

[7] Lu W, Du J, Goehring A, Gouaux E. Cryo-EM structures of the triheteromeric NMDA receptor and its allosteric modulation. Science. 2017;355(6331):eaal3729. 10.1126/science.aal3729 PMC5568803 28232581

[8] Karakas E, Furukawa H (2014). Crystal structure of a heterotetrameric NMDA receptor ion channel. Science..

[9] MacDonald JF, Porietis AV, Wojtowicz JM (1982). L-aspartic acid induces a region of negative slope conductance in the current-voltage relationship of cultured spinal cord neurons. Brain Res.

[10] Mendell LM (1966). Physiological properties of unmyelinated fiber projection to the spinal cord. Exp Neurol.

[11] Nowak L, Bregestovski P, Ascher P, Herbet A, Prochiantz A (1984). Magnesium gates glutamate-activated channels in mouse central neurones. Nature..

[12] Guerineau NC, Gahwiler BH, Gerber U (1994). Reduction of resting K+ current by metabotropic glutamate and muscarinic receptors in rat CA3 cells: mediation by G-proteins. J Physiol.

[13] Hayashi Y, Shi SH, Esteban JA, Piccini A, Poncer JC, Malinow R (2000). Driving AMPA receptors into synapses by LTP and CaMKII: requirement for GluR1 and PDZ domain interaction. Science..

[14] Sanhueza M, Fernandez-Villalobos G, Stein IS, Kasumova G, Zhang P, Bayer KU (2011). Role of the CaMKII/NMDA receptor complex in the maintenance of synaptic strength. J Neurosci.

[15] Malinow R, Schulman H, Tsien RW (1989). Inhibition of postsynaptic PKC or CaMKII blocks induction but not expression of LTP. Science..

[16] Silva AJ, Stevens CF, Tonegawa S, Wang Y (1992). Deficient hippocampal long-term potentiation in alpha-calcium-calmodulin kinase II mutant mice. Science..

[17] Carroll RC, Beattie EC, von Zastrow M, Malenka RC (2001). Role of AMPA receptor endocytosis in synaptic plasticity. Nat Rev Neurosci.

[18] Beattie EC, Carroll RC, Yu X, Morishita W, Yasuda H, von Zastrow M (2000). Regulation of AMPA receptor endocytosis by a signaling mechanism shared with LTD. Nat Neurosci.

[19] Mulkey RM, Endo S, Shenolikar S, Malenka RC (1994). Involvement of a calcineurin/inhibitor-1 phosphatase cascade in hippocampal long-term depression. Nature..

[20] Rose CR, Konnerth A (2001). NMDA receptor-mediated Na+ signals in spines and dendrites. J Neurosci.

[21] Yu XM, Salter MW (1998). Gain control of NMDA-receptor currents by intracellular sodium. Nature..

[22] Yu X-M, Groveman BR, Fang X-Q, Lin S-X (2010). The role of intracellular sodium (Na) in the regulation of calcium (Ca)-mediated signaling and toxicity. Health..

[23] Yu XM, Salter MW (1999). Src, a molecular switch governing gain control of synaptic transmission mediated by N-methyl-D-aspartate receptors. Proc Natl Acad Sci U S A.

[24] Xin WK, Kwan CL, Zhao XH, Xu J, Ellen RP, McCulloch CA (2005). A functional interaction of sodium and calcium in the regulation of NMDA receptor activity by remote NMDA receptors. J Neurosci.

[25] Nong Y, Huang Y-Q, Ju W, Kalia LV, Ahmadian G, Wang YT (2003). Glycine binding primes NMDA receptor internalization. Nature..

[26] Vissel B, Krupp JJ, Heinemann SF, Westbrook GL (2001). A use-dependent tyrosine dephosphorylation of NMDA receptors is independent of ion flux. Nat Neurosci.

[27] Nong Y, Huang YQ, Salter MW (2004). NMDA receptors are movin’ in. Curr Opin Neurobiol.

[28] Mugnaini M, Antolini M, Corsi M, van Amsterdam FT (1998). [3H]5,7-dichlorokynurenic acid recognizes two binding sites in rat cerebral cortex membranes. J Recept Signal Transduct Res.

[29] Mugnaini M, Dal Forno G, Corsi M, Bunnemann B (2000). Receptor binding characteristics of the novel NMDA receptor glycine site antagonist [3H]GV150526A in rat cerebral cortical membranes. Eur J Pharmacol.

[30] Popik P, Lewin A, Berrang B, Nowak G, Layer R, Skolnick P (1995). [3H]1-aminocyclopropanecarboxylic acid, a novel probe for strychnine- insensitive glycine receptors. Eur J Pharmacol.

[31] Colquhoun D (1998). Binding, gating, affinity and efficacy: the interpretation of structure-activity relationships for agonists and of the effects of mutating receptors. Br J Pharmacol.

[32] Danysz W, Parsons CG (1998). Glycine and N-methyl-D-aspartate receptors: physiological significance and possible therapeutic applications. Pharmacol Rev.

[33] Papouin T, Ladepeche L, Ruel J, Sacchi S, Labasque M, Hanini M (2012). Synaptic and extrasynaptic NMDA receptors are gated by different endogenous coagonists. Cell..

[34] Ferreira JS, Papouin T, Ladepeche L, Yao A, Langlais VC, Bouchet D, et al. Co-agonists differentially tune GluN2B-NMDA receptor trafficking at hippocampal synapses. Elife. 2017;6:e25492. 10.7554/eLife.25492 PMC5466419 28598327

[35] Fang X-Q, Qiao H, Groveman BR, Feng S, Pflueger M, Xin W-K (2015). Regulated internalization of NMDA receptors drives PKD1-mediated suppression of the activity of residual cell-surface NMDA receptors. Molecular Brain..

[36] Yu X-M, Fang X-Q, Jiang X-H. NMDA receptor internalization down-regulates NMDA receptor-mediated synaptic responses through the inhibition of remaining (non-internalized) surface NMDA receptors. Neurotransmitter. 2016;3:e1192.

[37] Nabavi S, Kessels HW, Alfonso S, Aow J, Fox R, Malinow R (2013). Metabotropic NMDA receptor function is required for NMDA receptor-dependent long-term depression. Proc Natl Acad Sci U S A.

[38] Stein IS, Gray JA, Zito K (2015). Non-Ionotropic NMDA receptor signaling drives activity-induced dendritic spine shrinkage. J Neurosci.

[39] Zhu JJ, Qin Y, Zhao M, Van Aelst L, Malinow R (2002). Ras and rap control AMPA receptor trafficking during synaptic plasticity. Cell..

[40] Eales KL, Palygin O, O'Loughlin T, Rasooli-Nejad S, Gaestel M, Muller J (2014). The MK2/3 cascade regulates AMPAR trafficking and cognitive flexibility. Nat Commun.

[41] Malenka RC, Bear MF (2004). LTP and LTD: an embarrassment of riches. Neuron..

[42] Tamburri A, Dudilot A, Licea S, Bourgeois C, Boehm J (2013). NMDA-receptor activation but not ion flux is required for amyloid-beta induced synaptic depression. PLoS One.

[43] Kessels HW, Nabavi S, Malinow R (2013). Metabotropic NMDA receptor function is required for -amyloid-induced synaptic depression. Proc Natl Acad Sci.

[44] Birnbaum JH, Bali J, Rajendran L, Nitsch RM, Tackenberg C (2015). Calcium flux-independent NMDA receptor activity is required for Abeta oligomer-induced synaptic loss. Cell Death Dis.

[45] Weilinger NL, Tang PL, Thompson RJ (2012). Anoxia-induced NMDA receptor activation opens Pannexin channels via Src family kinases. J Neurosci.

[46] Weilinger NL, Lohman AW, Rakai BD, Ma EMM, Bialecki J, Maslieieva V (2016). Metabotropic NMDA receptor signaling couples Src family kinases to pannexin-1 during excitotoxicity. Nat Neurosci.

[47] Han L, Campanucci VA, Cooke J, Salter MW (2013). Identification of a single amino acid in GluN1 that is critical for glycine-primed internalization of NMDA receptors. Molecular Brain.

[48] Han L (2012). Molecular mechanisms of Glycine primed NMDA receptor internalization [thesis (Ph D )]: University of Toronto.

[49] Sengar AS, Li H, Zhang W, Leung C, Ramani AK, Saw NM (2019). Control of long-term synaptic potentiation and learning by alternative splicing of the NMDA receptor subunit GluN1. Cell Reports.

[50] Li LJ, Hu R, Lujan B, Chen J, Zhang JJ, Nakano Y (2016). Glycine potentiates AMPA receptor function through metabotropic activation of GluN2A-containing NMDA receptors. Front Mol Neurosci.

[51] Otsu Y, Darcq E, Pietrajtis K, Matyas F, Schwartz E, Bessaih T (2019). Control of aversion by glycine-gated GluN1/GluN3A NMDA receptors in the adult medial habenula. Science..

[52] Dore K, Aow J, Malinow R (2015). Agonist binding to the NMDA receptor drives movement of its cytoplasmic domain without ion flow. Proc Natl Acad Sci U S A.

[53] Esmenjaud JB, Stroebel D, Chan K, Grand T, David M, Wollmuth LP, et al. An inter-dimer allosteric switch controls NMDA receptor activity. EMBO J. 2019;38(2):e99894. 10.15252/embj.201899894 PMC6331725 30396997

[54] Montes de Oca Balderas P, Aguilera P (2015). A Metabotropic-Like Flux-Independent NMDA Receptor Regulates Ca2+ Exit from Endoplasmic Reticulum and Mitochondrial Membrane Potential in Cultured Astrocytes. PLoS One.

[55] Gerard F, Hansson E (2012). Inflammatory activation enhances NMDA-triggered Ca2+ signalling and IL-1beta secretion in primary cultures of rat astrocytes. Brain Res.

[56] Standley S, Roche KW, McCallum J, Sans N, Wenthold RJ (2000). PDZ domain suppression of an ER retention signal in NMDA receptor NR1 splice variants. Neuron..

[57] Morise J, Suzuki KGN, Kitagawa A, Wakazono Y, Takamiya K, Tsunoyama TA (2019). AMPA receptors in the synapse turnover by monomer diffusion. Nat Commun.

